# Supplementary material for: Role and mechanism of NCAPD3 in promoting malignant behaviors in gastric cancer
Source: Front Pharmacol. 2024 Apr 22;15:1341039. doi: 10.3389/fphar.2024.1341039 (PMC11070777; doi:10.3389/fphar.2024.1341039)
Supplement: Supplementary file 11 [file DataSheet2.ZIP › GSEA/GO_CC/my_analysis.Gsea.1599461408869/GO_BASOLATERAL_PLASMA_MEMBRANE.html]

Details for gene set GO\_BASOLATERAL\_PLASMA\_MEMBRANE[GSEA]

|  || Dataset | filtered\_dataset.sample\_info.cls#WT\_versus\_NCAPD3\_MUT |
| Phenotype | sample\_info.cls#WT\_versus\_NCAPD3\_MUT |
| Upregulated in class | NCAPD3\_MUT |
| GeneSet | GO\_BASOLATERAL\_PLASMA\_MEMBRANE |
| Enrichment Score (ES) | -0.32473686 |
| Normalized Enrichment Score (NES) | -1.5654576 |
| Nominal p-value | 0.04404145 |
| FDR q-value | 0.118124574 |
| FWER p-Value | 0.818 |
Table: GSEA Results Summary

  

Fig 1: Enrichment plot: GO\_BASOLATERAL\_PLASMA\_MEMBRANE      
 Profile of the Running ES Score & Positions of GeneSet Members on the Rank Ordered List

  

| SYMBOL | TITLE | RANK IN GENE LIST | RANK METRIC SCORE | RUNNING ES | CORE ENRICHMENT || 1 | 659 | BMPR2 | 16 | 1.047 | 0.0639 | No |
| 2 | 84976 | DISP1 | 221 | 0.659 | -0.0355 | No |
| 3 | 6573 | SLC19A1 | 231 | 0.650 | 0.0049 | No |
| 4 | 538 | ATP7A | 305 | 0.588 | -0.0053 | No |
| 5 | 960 | CD44 | 339 | 0.573 | 0.0122 | No |
| 6 | 8573 | CASK | 368 | 0.554 | 0.0320 | No |
| 7 | 5311 | PKD2 | 708 | 0.372 | -0.1854 | No |
| 8 | 153562 | MARVELD2 | 732 | 0.359 | -0.1761 | No |
| 9 | 1605 | DAG1 | 823 | 0.304 | -0.2190 | No |
| 10 | 7037 | TFRC | 824 | 0.303 | -0.1971 | No |
| 11 | 360 | AQP3 | 857 | -0.263 | -0.2012 | No |
| 12 | 667 | DST | 886 | -0.303 | -0.1995 | No |
| 13 | 8140 | SLC7A5 | 890 | -0.305 | -0.1797 | No |
| 14 | 301 | ANXA1 | 914 | -0.342 | -0.1716 | No |
| 15 | 3949 | LDLR | 929 | -0.353 | -0.1562 | No |
| 16 | 9076 | CLDN1 | 1084 | -0.463 | -0.2337 | No |
| 17 | 9962 | SLC23A2 | 1169 | -0.537 | -0.2556 | No |
| 18 | 1956 | EGFR | 1266 | -0.657 | -0.2773 | Yes |
| 19 | 286 | ANK1 | 1284 | -0.672 | -0.2411 | Yes |
| 20 | 4233 | MET | 1358 | -0.821 | -0.2345 | Yes |
| 21 | 9990 | SLC12A6 | 1379 | -0.922 | -0.1824 | Yes |
| 22 | 493 | ATP2B4 | 1401 | -1.166 | -0.1135 | Yes |
| 23 | 760 | CA2 | 1410 | -1.654 | -0.0000 | Yes |
Table: GSEA details [plain text format]

  

Fig 2: GO\_BASOLATERAL\_PLASMA\_MEMBRANE      
 Blue-Pink O' Gram in the Space of the Analyzed GeneSet

  

Fig 3: GO\_BASOLATERAL\_PLASMA\_MEMBRANE: Random ES distribution      
 Gene set null distribution of ES for **GO\_BASOLATERAL\_PLASMA\_MEMBRANE**
